# 

**Published:** 1871-07

**Authors:** 


### CONTENTS:

ORIGINAL COMMUNICATIONS:

ARTICLE I. On the Use of Carbolic Acid in Diptheritic Affections.—By F. C. Hotz, M.D. .... 385

ART. II. The Microscopic Appearances of Cancer Cells. By I. N. Danforth, M.D. .... 391

ART. III. On a few Instruments for The Practical treatment of Uterine Disease. By Philip Adolphus, M.D. (Fourth Paper) .... 398

ART. IV. Delivery of Extra-uterine Fœtus through the Abdominal Walls after four Years. Reported by J. H. Beech, M.D .... 406

ART. V. Case of Sub-acute Colitis, with Suppuration. By James S. Whitmire, M.D .... 409

ART. VI. Case of Hepatic Abscess, involving the Gall Bladders, successfully treated by injections of Solutions of Iodine. By J. A. Goldsberry, M.D .... 412

ART. VII. Hypodermic Medication. By T. Curtis Smith, M.D .... 418

ART. VIII. Clinical Lectures on Diseases of the Ear. By E. L. Holmes, M.D .... 436

EDITOR'S BOOK TABLE .... 440

EDITORIAL .... 445

PROCEEDINGS OF SOCIETIES .... 447

